# Discovering the Effects of Superior-Surface Vocal Fold Lesions via Fluid–Structure Interaction Analysis

**DOI:** 10.3390/bioengineering12121360

**Published:** 2025-12-13

**Authors:** Manoela Neves, Anitha Niyingenera, Norah Delaney, Rana Zakerzadeh

**Affiliations:** Department of Biomedical Engineering, School of Science and Engineering, Duquesne University, Pittsburgh, PA 15219, USA

**Keywords:** vocal polyps, lesions, fluid–structure interaction (FSI), computational modeling and simulation, vocal fold vibration, glottal aerodynamics, phonation biomechanics

## Abstract

This study examines the impact of vocal fold (VF) lesions located on the superior surface on glottal airflow dynamics and tissue oscillatory behaviors using biomechanical simulations of a two-layered realistic VF model. It is hypothesized that morphological changes in the VFs due to the presence of a lesion cause changes in tissue elasticity and rheological properties, contributing to dysphonia. Previous research has lacked the integration of lesions in computational simulations of anatomically accurate larynx-VF models to explore their effects on phonation and contribution to voice disorders. Addressing the current gap in literature, this paper considers a computational model of a two-layered VF structure incorporating a lesion that represents a hemorrhagic polyp. A three-dimensional, subject-specific, multilayered geometry of VFs is constructed based on STL files derived from a human larynx CT scan, and a fluid–structure interaction (FSI) methodology is employed to simulate the coupling of glottal airflow and VF tissue dynamics. To evaluate the effects of the lesion’s presence, two FSI models, one with a lesion embedded in the cover layer and one without, are simulated and compared. Analysis of airflow dynamics and tissue vibrational patterns between these two models is used to determine the impact of the lesion on the biomechanical characteristics of phonation. The polyp is found to slightly increase airflow resistance through the glottis and disrupt vibratory symmetry by decreasing the vibration frequency of the affected fold, leading to weaker and less rhythmic oscillations. The results also indicate that the lesion increases tissue stress in the affected fold, which agrees with clinical observations. While quantitative ranges depend on lesion size and tissue properties, these consistent and physically meaningful trends highlight the biomechanical mechanisms by which lesions influence phonation.

## 1. Introduction

Human phonation arises from the intricate interaction between airflow passing through the larynx and the vibrations of the vocal folds (VFs) induced by this flow. Prolonged or excessive exposure to such vibrations is recognized as an important risk factor contributing to the development of VF pathologies and resulting in alterations of the tissue’s mechanical characteristics [1,2]. Clinically, phono-trauma manifests in a few different forms, one of which is the formation of lesions [3].

Vocal lesions are characterized by the growth of abnormal tissue within the vibratory layer of the fold [4], swelling [5], or the rupture of dilated blood vessels under the epithelium cover [6]. These lesions vary in form (polyps, cysts, nodules, vascular lesions, and Reinke’s edema are a few common variations), size, and biomechanical features, suggesting that their impact on voice production may differ [5]. Their presence weighs down the VFs and leads to changes in tissue elasticity [7], which in turn interrupts the oscillation [8], potentially causing dysphonia [9], progressive development of voice fatigue [10], and decreased voice quality [11].

Clinical studies had a lively interest in studying the influence of various VF lesions on voice quality, acoustic analysis of patients [12], and management modalities [13], demonstrating that even minor alterations in VF morphology significantly impact phonatory efficiency and acoustic outcomes [14]. However, despite the established role of VF lesions on voice characteristics, limited basic research has been performed to elucidate how these lesions affect fundamental processes of phonation and, therefore, speech biomechanics. The impact of geometric abnormality due to an in vitro VF polyp model on glottal jet behavior was investigated by [15] using particle image velocimetry (PIV) measurements, while [16] examined the impact of nodule size and stiffness by placing spherical lesions at the surface of synthetic VF replicas, in a hemilaryngeal flow facility. Although clinical assessments and experimental methods provide valuable insights, computational modeling offers a crucial approach to investigating the biomechanical disruptions caused by these lesions.

In VF research, numerical methodologies such as computational fluid dynamics (CFD) as well as fluid–structure interaction (FSI) simulations have been used extensively to explore complex dynamics of glottal airflow and VF tissue function [17,18,19,20,21,22]. Very few computational studies have focused on the function of VFs under pathological conditions [23,24,25,26,27,28], and none of them have incorporated the presence of lesions in FSI simulations. The study by [23] explored the effects of small and large VF lesions on the particle deposition pattern using CFD. However, the effect of VF lesions was mimicked by changing the glottal width, assuming stationary VFs and VF lesions. Ref. [24] developed a mathematical model to examine the relation between intravascular pressure and VF vibration with consideration of benign lesions. Jiang and colleagues [27] developed a computer-based model to analyze the vibrating modes of the VFs in the presence of a nodule, in normal phonation and hyperfunctional dysphonia. However, the vibrations were generated by applying intraglottal pressures directly to the fold surfaces, which does not capture the realistic physics of phonation because it ignores the fully coupled interaction between airflow and tissue. In a later study, ref. [28] evaluated the unstable vibration patterns of the folds in the presence of a unilateral polyp, with a simplified two-mass model that does not represent the folds and vocal tract accurately, and neglected FSI features.

**Figure 1:**
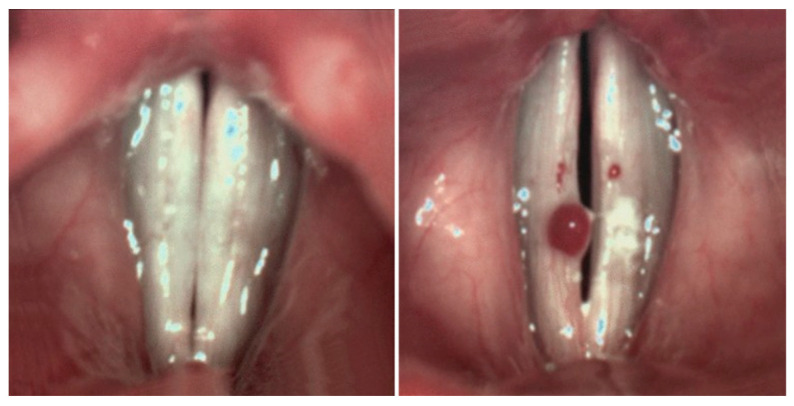
Comparison of glottal images for a normal VF case (**left**) and a VF with a unilateral hemorrhagic polyp (**right**), adapted from [32] with permission under a Creative Commons Attribution (CC BY) license.

Given the gap in existing research, the aim of this study is to evaluate the contribution of these lesions on aerodynamics and flow-induced vibrations of VF tissue using FSI simulations. In this paper, for the first time, a computer simulation tool has been utilized to obtain the contribution of benign lesions to glottal flow and tissue vibratory response in a patient-specific VFs model. While the framework is general, we develop the model by considering the lesion as a hemorrhagic VF polyp (VFP) shown in Figure 1. VFPs are considered the second most prevalent laryngeal lesion after nodules [29]. Polyps can be unilateral or bilateral [30] but are typically unilateral. Often, there will be a hemorrhagic nature to the polyp, giving it a maroon or blood-colored appearance [31].

## 2. Methods: Formulation and Procedure

### 2.1. Geometry

**Figure 2:**
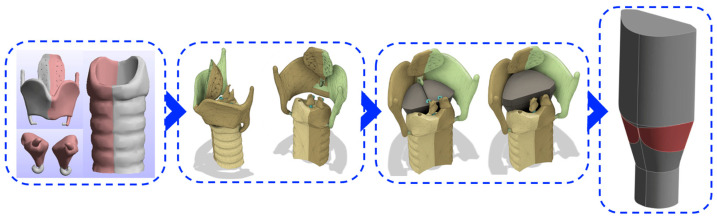
The process of constructing the vocal tract and subject-specific VF model using STL files of a human laryngeal CT scan. The folds are positioned within the laryngeal area based on the locations of relevant cartilages, and the air domain is extruded from the VF structure.

The geometry of the vocal tract is reconstructed from the features of a laryngeal CT scan of a human subject retrieved from the NIH 3D repository [33]. Using three STL files of cartilages, (thyroid cartilage and epiglottis, cricoid and tracheal cartilage, right and left arytenoid cartilage), an anatomically consistent transverse cross-section of the VFs is approximated by defining the contours according to the spatial relationships and geometric constraints provided by the assembled cartilages. Using this outline, two roughly triangular-shaped bands representing the VF tissue are created (Figure 2). The appropriate morphological dimensions were sketched directly from the thyroid cartilage piece and the two bands of the VFs were formed with the glottal gap positioned relative to the arytenoid cartilage piece. The 2D cross-section of the VFs was then extruded to generate the 3D model. The Autodesk Fusion 360 software is used to segment the air lumen and divide the VF domains. The lumen is extruded to create subglottal and supraglottal regions. The VFs are located between 2.5 cm and 3.4 cm from the bottom inlet in the inferior-superior direction.

**Figure 3:**
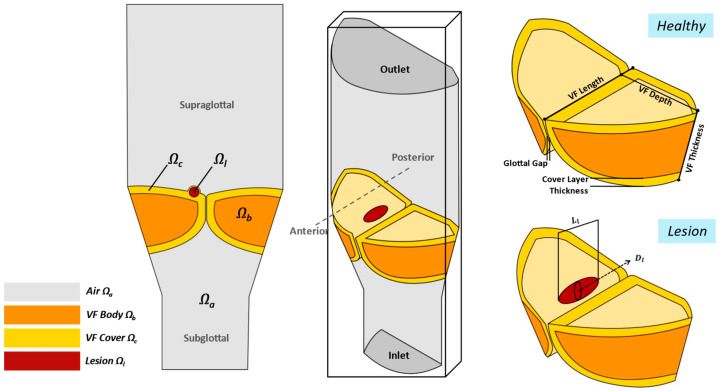
Schematic of the larynx and VFs domains, highlighting their geometric features.

The length, thickness, and depth of the folds are illustrated in Figure 3. The corresponding values presented in Table 1 are consistent with the geometrical features of human subject models in [19,21,34], and remain within the established minimum and maximum limits reported for male and female morphology for each parameter as listed in Table I of [18]. The VF is a multilayer structure that includes a cover layer (i.e., epithelium and superficial lamina propria) that is composed of elastin and collagen fibers, and a body layer that consists of thyroarytenoid muscle. Therefore, the structure of the VF tissue is divided into cover and body sub-layers to reflect the differences in biomaterial properties. The cover layer is created by offsetting the superior, medial, and inferior VF surfaces by 0.1 cm in thickness, consistent with values in [20,35].

A geometric protuberance representing a VFP is added to the left VF geometry. This lesion is placed in the middle of the VF along the anteroposterior direction, adjacent to the glottal gap, and situated on the superior surface. It is embedded within the cover layer [29]. Given the lack of precise data on lesion sizes, the dimensions are selected based on prior studies [36,37], and clinical observations that most VFPs are larger than 0.3 cm [38]. Previous literature has observed that VF lesions can range from 0.1 to 0.6 cm in length [12,16,39]. Therefore, the considered lesion has an elliptical shape with a length of 0.37 cm and a diameter of 0.15 cm, approximating one-third of the total VF length. A schematic overview of the model under consideration is displayed in Figure 3. An elliptical lesion geometry provides a reasonable approximation of typical benign VFPs shape, in accordance with prior studies [15,40] and clinical descriptions of polyps as rounded or oblong protrusions [29,41], making it an appropriate generic representation.

### 2.2. Governing Equations of Airflow, VFs Tissue, and Lesion

The three domains, larynx, VFs, and lesion, are meshed in ANSYS Meshing using tetrahedral and hexahedral elements. Two separate models are created: one incorporates a VFP in the left fold, while the other maintains the same geometry of the laryngeal air domain but without the lesion.

The glottal airflow in the larynx $\Omega_{a}(t)$ is modeled by viscous, incompressible Navier-Stokes Equations (1) and (2):

(1) $$\rho_{a}\left(\frac{\partial v}{\partial t}+v\cdot \nabla v\right)=\nabla \cdot \sigma_{a}$$

(2) ∇·v=0

where $U_{a}$ is the velocity, $\sigma_{a}=-p_{a}I+2\mu_{a}D(U_{a})$ represents the fluid stress tensor, $p_{a}$ indicates the fluid pressure, $\rho_{a}$ is the fluid density,$\mu_{a}$ is the fluid viscosity, and $D\left(U_{a}\right)=\frac{1}{2}(\nabla U_{a}+\nabla {U_{a}}^{T})$ is the strain rate tensor. The air density is taken to be ${\;\rho }_{a}$ = 1.185 kg/m^3^ and viscosity $\mu_{a}=$ 1.83 × 10^−5^ kg/m.s, representing properties at 25 °C temperature. Airflow is also considered to be laminar, which provides a reasonable approximation for laryngeal flow as discussed in [42], and is acceptable for modeling FSI in VFs, similar to many previous models [17,26,34,35,43].

The VFs’ structure is represented as a composite material consisting of a body layer and a cover layer. Hence, the deformation of the cover/body complex is modeled using the elastodynamic equations of motion (3), solved individually for each layer:

(3) $$\nabla \rho_{cb}\frac{D^{2}U_{cb}}{Dt^{2}}=\nabla .\left(\sigma_{s}\right)$$

where $U_{cb}$ is the VF structure displacement and $\rho_{cb}$ is the density. Both layers have the same density of $\rho_{b}$ = $\rho_{c}$ = 1070 kg/m^3^, that is reasonably expected to lie within the range of density for human tissues, which has been reported to be 950–1100 kg/m^3^. One of the common simplifying assumptions in models of airflow interaction with the VFs tissue is the treatment of the biomechanical material properties of the VFs as linearly elastic, homogeneous material [44,45]. Some authors claim that although human VF tissue acts as a non-linear material, during phonation under active muscular tension the VFs display an approximately linear stress–strain relationship [46,47,48]. Hence, we assume that the tissue behaves as an isotropic, linearly elastic Saint-Venant Kirchhoff material. It should be noted that although biological tissues exhibit nonlinearity and viscoelasticity, modeling VFs using a linear elastic constitutive law has been widely used in previous phonation modeling studies such as [26,43,49,50] to reduce computational complexity in the two-way FSI simulations. Therefore,

(4) $$\sigma_{s}=\frac{E_{cb}}{1+\mu_{cb}}D\left(U_{cb}\right)+\frac{E_{cb}\mu_{cb}}{\left(1+\mu_{cb})(1-2\mu_{cb}\right)}(\nabla \cdot U_{cb})I$$

where $E_{cb}$ and $\mu_{cb}$ are the Young’s modulus and the Poisson’s ratio, respectively. Studies have measured the Young’s moduli for the tissues to be in the range between 3.9 kPa and 110 kPa [51]. The body elastic modulus is assumed to be 4 times higher than that of the cover, $E_{c}$= 10 kPa and $E_{b}$= 40 kPa, supported by similar studies [26,35]. Poisson’s ratio of both layers is set to $\mu_{cb}$ = 0.45 [52]. The tissue deformation gradient is $D\left(U_{cb}\right)=\frac{1}{2}(\nabla U_{vf}+\nabla {U_{vf}}^{T})$, defined under the assumption of small deformations.

The VFP lesion deformations are described by the momentum equation for balance of total forces (5):

(5) $$\rho_{l}\frac{D^{2}U_{l}}{Dt^{2}}=\nabla .\left(\sigma_{l}\right)$$

where $U_{l}$ is the displacement, $\rho_{l}$ represents the lesions’ density and $\sigma_{l}$ denotes the elasticity stress tensor for the Saint Venant-Kirchoff elastic material,

(6) $$\sigma_{l}=\frac{E_{l}}{1+\mu_{l}}D\left(U_{l}\right)+\frac{E_{l}\mu_{l}}{\left(1+\mu_{l})(1-2\mu_{l}\right)}(\nabla \cdot U_{l})I$$

where, $E_{l}$ and $\mu_{l}$ are the Young’s modulus and the Poisson’s ratio of the lesion tissue, respectively. Considering the lesion as a hemorrhagic polyp, the region is filled with clotted blood. Therefore, we used the value of $\rho_{l}$ = 1300 kg/m^3^ based on the measurements from [53]. The physiological range of lesion elastic moduli is not well known, although lesions are typically stiffer than the surrounding VF tissue [54]. We assume $E_{l}$ = 5000 kPa and $\mu_{l}$ = 0.49 obtained from the experimental measurements of blood clots reported in [55], and comparable with the elasticity value adopted previously in [27].

### 2.3. Boundary Conditions and FSI Setup

**Figure 4:**
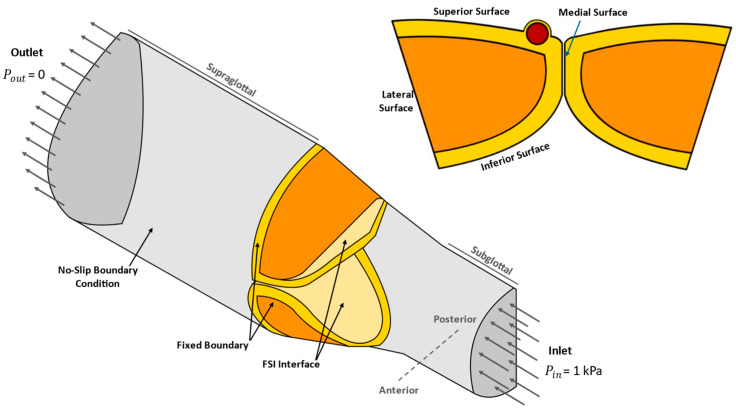
Illustration of the applied boundary conditions and the FSI interface.

The inflow and outflow boundaries are located at the subglottal and supraglottal ends of the vocal tract channel, as shown in Figure 4. A physiologically realistic pressure value of $P_{in}$= 1 kPa, corresponding to 10.2 cm of water, at the inlet is enforced. This value is in agreement with the recorded glottal air pressure data for the normal speaking condition in [56], and consistent with measurements by [57]. The supraglottal gauge pressure is prescribed as $P_{out}$ = 0 Pa at the outlet. The no-slip boundary condition is applied on all other bounding surfaces of the fluid domain except for the inlet and outlet. The surfaces of the folds exposed to airflow are free to move, whereas the remaining boundaries are held fixed. Specifically, a fixed boundary condition is imposed on the anterior, posterior, and lateral surfaces of the VFs cross sections that are in direct contact with the cartilage structures. The interaction between the tissue and the airflow is defined along the VFs’ superior, medial, and inferior surfaces identified by the FSI interface (Figure 4). The kinematic (no-slip) and dynamic (continuity of the normal stresses) coupling conditions are applied at these shared flow-tissue interfaces.

To solve Equations (1)–(6) together, the FSI techniques developed in our previous studies [18,22] are utilized. The implementation of the FSI solver for an idealized healthy VF (without the lesion) model and the numerical simulation settings are outlined comprehensively in [58], while some details are given in Section 2.4. In this study, we have extended the methodology for a multilayered VF structure located within a patient-specific larynx geometry, which needs to consider the biomechanical properties of cover and body tissues, the contact between two folds, and the contact between the fold and lesion tissue. These features are implemented in ANSYS Workbench software (version 2024 R2, ANSYS Inc., Canonsburg, PA, USA) platform. The ANSYS System Coupling module [59] specifies the behavior of the FSI interface by coordinating the exchange of solution data between the CFX and Mechanical components of the software. The CFX component is used to calculate aerodynamics of the glottal flow within the larynx, while the Mechanical component is used to solve for structural deformations of the VF and lesion tissues during phonation. Execution and convergence of airflow and tissue simulations are governed by the System Coupling component, which handles the fluid and structural domains in a sequential, independent manner.

To study the effect of the lesion, we perform FSI simulations of two cases: one case of healthy VFs and a case of a lesion as shown in Figure 3. The selected final mesh for the lesion case model is split into 1.7 million quadratic and tetrahedral elements within the larynx, lesion, and VF regions. The computational model features a non-uniform discretization, providing enhanced resolution in the intraglottal region due to the anticipated large flow gradients, as well as a more detailed grid and increased mesh refinement with tetrahedral elements in the groove areas and near the FSI interface of the VFs. A quadratic mesh is used in the subglottal and supraglottal regions, where the geometry is more linearly structured. The mesh convergence study is conducted to ensure an optimally sized mesh density that would give an accurate converged solution at an optimal computational time, as described in Section 2.4, similar to the approach in prior work [58]. A sufficient number of cycles is simulated until steady-state vibration is reached.

### 2.4. Computational Modeling Details

The parameters, conditions, and governing equations described in Section 2.1, Section 2.2 and Section 2.3 were implemented using ANSYS Workbench (version 2025.R1, ANSYS Inc., Canonsburg, PA, USA) to perform fully coupled transient FSI simulations. The aerodynamic behavior was modeled with the CFD solver in the ANSYS CFX component, employing the finite volume method (FVM), whereas the structural deformations of the VF tissue during phonation were simulated in ANSYS Mechanical using the finite element method (FEM). Interaction between the airflow and VFs structures occurs via a single fluid–solid interface, which is managed by the ANSYS System Coupling module, verified and validated in [59].

For the two-way FSI simulations, two types of data transfers are executed. The first transfer conveys the fluid forces calculated by the CFD solver at the shared airflow–tissue interface to the corresponding interface in the Mechanical solver. The second transfer communicates the incremental displacements from the Mechanical solver back to the air domain, applying them as mesh displacements in the CFD solver. The ANSYS System Coupling module supervises both the execution and convergence of the coupled simulation, solving the fluid and solid domains. More precisely, the airflow simulation is performed in ANSYS CFX until the specified residual convergence is met, providing the pressure distribution and velocity fields within the larynx, along with the forces acting on the FSI interface. These forces are then applied in ANSYS Mechanical to compute the resulting VF tissue deformations, producing a converged displacement solution. The updated displacements are then sent back to the CFX solver, and this iterative procedure continues for the number of defined coupling steps. For each time step, a maximum of ten coupling iterations is allowed to achieve convergence, which is confirmed when the root-mean-square residual reaches 0.01. The staggered iterations are restarted with the updated mesh generated by solid deformation.

Mesh deformation within CFX is solved using a displacement diffusion equation to propagate displacements throughout the computational domain. A locally increased mesh stiffness (i.e., adjusted diffusivity for the mesh displacement equation) is applied to maintain element quality and prevent mesh folding in regions experiencing large deformation. The solution from the previous step is interpolated onto the updated mesh using the tools provided by ANSYS-CFX. As for the FSI boundary conditions, the displacement of the interface Γ is assumed to be equal for both fluid and solid domains, enforcing the adhesion of the fluid to the tissue. Normal stress equilibrium is also applied along the interface. The System Coupling module ensures that the coupling conditions, namely the continuity of velocities and normal stresses between fluid and tissue, are satisfied. Force and displacement data exchange occurs at the start of each coupling iteration within a step. The Conservative Profile Preserving algorithm is used for force transfer, while the General Grid Interface (GGI) mapping algorithm calculates the appropriate mapping weights. Displacements are transferred using the Profile Preserving algorithm in combination with the Bucket Surface mapping approach, accommodating any mismatched meshes at the interface. The procedures for data transfer and mesh deformation are described in detail in [58]. Convergence criteria in ANSYS CFX are defined as 10^−5^ for the normalized residuals of the global linear system for mass and momentum. Convective terms in the Navier–Stokes equations are linearized using Picard iterations, with pressure evaluated at the same nodes as velocity. The Laplace operator in the momentum equations is approximated via a centered scheme, and convective terms are discretized using a second-order upwind scheme. The resulting linear system is solved using an algebraic multigrid method with incomplete LU factorization as a smoother.

Contact between the VFs is modeled using the Augmented Lagrange method, a penalty-based approach in ANSYS Mechanical. This approach computes contact forces based on stiffness and penetration, similar to the Pure Penalty method, but enhanced with a Lagrange multiplier to improve convergence. Mesh sensitivity analysis follows the methodology described in our previous work [58]. In this process, the element count is progressively increased, and simulations are repeated on successively finer grids until the resulting predictions show close agreement. Three meshes, containing approximately 1.2, 1.7, and 2.1 million elements, were tested. For the vocal fold (VF) region, the mesh is deemed adequate when further refinement leads to no substantial change in the maximum VF displacement, corresponding to a relative difference below 5%. Moreover, a comparison between medium and fine meshes indicates minimal discrepancy (relative error less than 7%) when evaluating the peak glottal gap width and the overall displacement profiles at selected locations. Similarly, the fluid domain is considered mesh-independent when airflow velocity and pressure variations within the glottis remain below a 5% threshold. Therefore, the medium-resolution grid with 1.7 million elements was selected for the simulations.

## 3. Simulation Results

**Figure 5:**
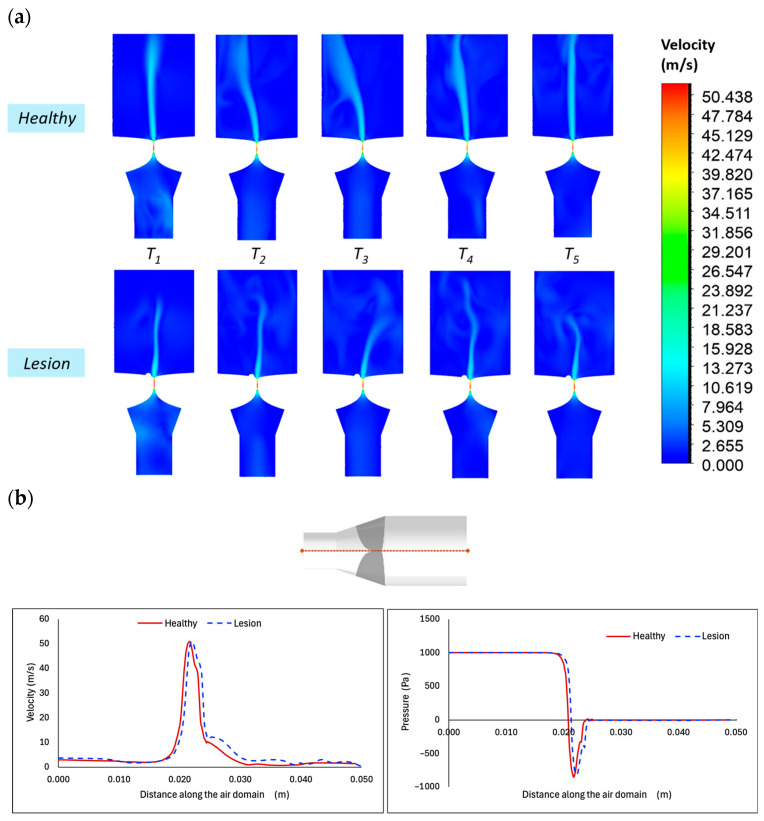
(**a**) Airflow velocity contours on the mid-coronal plane for healthy VFs (**top**) and VFs with a polyp (**bottom**) at five time points representing different phases of an oscillation cycle. (**b**) A comparison of intraglottal airflow velocity and axial pressure distribution in the glottal area obtained for cases with and without a lesion, captured at the glottal opening time instant. Path line is shown at the top with red.

Predictions for laryngeal aerodynamics and VF tissue deformations between a diseased and a healthy VF model, when the VFP is excluded, are compared to identify how the lesion influences the results. Aerodynamic data are plotted in Figure 5.

The velocity contours within the vocal tract at five distinct time steps, each spaced 0.02 s apart during a phonation cycle, are visualized in Figure 5a. The narrowed glottic area leads to a pressure drop and a corresponding increase in velocity, with the peak occurring within the glottis. In both cases, regardless of the presence of a lesion, vortices are observed above the glottis and persist into the supraglottal region. However, polyp-induced asymmetry disrupts vortex formation and alters the recirculation zones. In the lesioned case, the jet decreases in both diameter and length, indicating a weaker flow, while the healthy case exhibits greater jet diffusion. Figure 5b compares velocity and pressure in the larynx for normal VFs and those with a polyp, measured along the centerline at the time point $T_{3}$ = 0.035 s, corresponding to the opening phase of the oscillation cycle (as shown in Figure 5a). Overall, velocity and pressure values remain comparable between cases. However, in the diseased case, peak velocity and pressure drop occur further downstream, indicating a noticeable lag in flow development relative to the healthy configuration. Compared to the lesioned case, the healthy VF case shows a lower dip in the pressure and a slightly higher glottal velocity, demonstrating reduced flow resistance.

**Figure 6:**
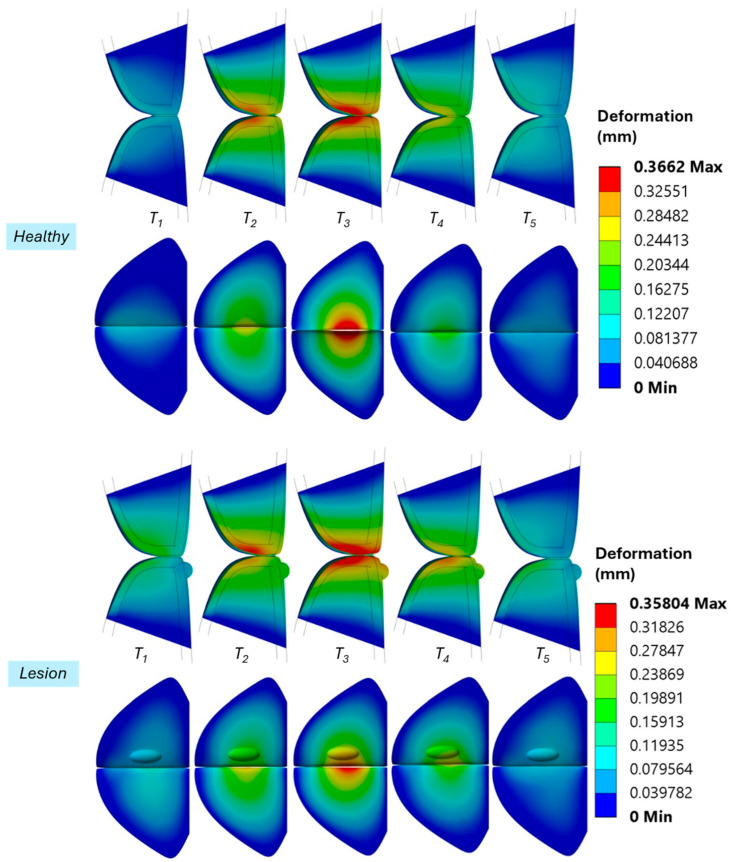
Deformation of the VFs in the healthy case and the polyp case during phonation, colored by total displacement. Snapshots progress from opening to closure. The first row presents mid-coronal profiles, while the second row shows superior views.

Figure 6 presents VF total displacement profiles at five time points within a single phonation cycle for both healthy and lesioned models, evaluated on the superior and mid-coronal planes. These snapshots correspond to the timesteps shown in Figure 5a. Both cases exhibit rhythmic VF vibrations, with the cycle progressing from full closure to maximum opening and back to closure. While the overall deformation magnitudes are similar, the case with VFP lesion exhibits pronounced asymmetry, with one VF consistently showing greater displacement, highlighted by the warmer color regions. This asymmetry is most evident at time point $T_{3}$, corresponding to the maximum glottal opening phase. In contrast, the healthy model displays symmetrical, synchronous motion, with equivalent displacement in both folds. The deformation pattern in the lesion case reflects a phase delay in the affected fold compared to its counterpart.

**Figure 7:**
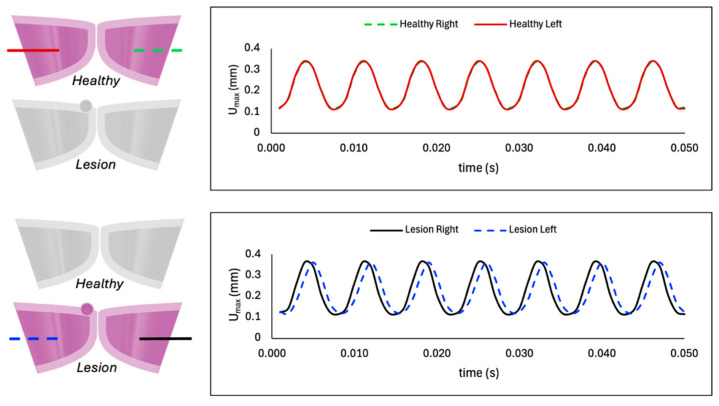
Maximum displacement over time for the healthy (**top**) and lesioned (**bottom**) models, comparing left and right folds in each case.

**Figure 8:**
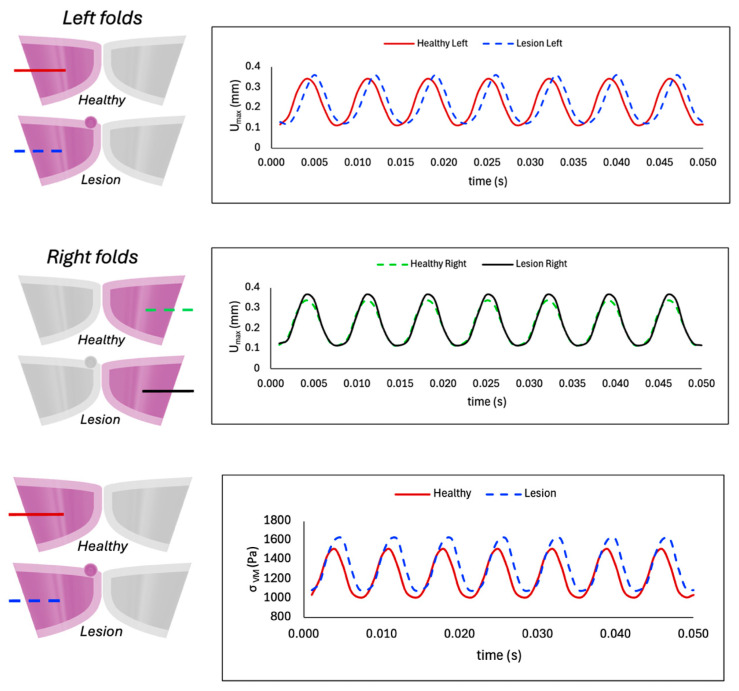
Comparison of maximum displacement over time between the right folds (**top panel**) and left folds (**middle panel**) of healthy and lesioned cases. The (**bottom panel**) shows maximum von Mises stress values for both cases.

Figure 7 compares the maximum displacement between the left and right folds in the healthy model and when a lesion is included. The top panel confirms synchronized, symmetric motion in the healthy case, with matched amplitude and frequency between folds. In contrast, the affected fold in the lesioned case vibrates with reduced amplitude and lower frequency (i.e., blue dashed line for lesion-left is slightly shifted to the right), indicating that the diseased fold has a weaker vibration than its opposing healthy one. Comparison of individual fold deformation between the healthy and lesioned cases in Figure 8 indicates that for the right fold (without VFP), the lesioned case exhibits a slightly increased amplitude, while both cases share the same vibration frequency and move in tandem. In contrast, for the left fold (with VFP), the lesioned model demonstrates a noticeable increase in amplitude but also a reduction in frequency, indicating slower vibration. The bottom panel of Figure 8 depicts von Mises stress in the left folds, where the lesioned fold displays significantly higher stress values and a lower frequency compared to the healthy fold.

## 4. Discussion

Studies using PIV [15,60] have shown that the presence of a polyp disrupts normal airflow behavior of the glottal jet throughout the phonatory cycle, leading to the development of vortical flow structures in the supraglottal region. These vortices are observed to be correlated with the energy exchange process that drives VF motion [60]. Such flow alterations can reduce aerodynamic efficiency, impacting vocal effort and quality. The findings in Figure 5a revealed significant disruptions in the airflow patterns in the presence of the polyp. The increased fluid jet diameter and diffusion in the healthy case compared with the lesion model (Figure 5a) affects the downstream recirculation zones and the produced vortices in the supraglottal region, while in the lesioned case, the flow jet and vortex structures are weaker. Additionally, polyp morphology alters the shape and contour of the glottal channel, resulting in modest increased airflow resistance through the glottis (Figure 5b).

As shown in Figure 6 and Figure 7, the diseased case largely mirrors the healthy model in vibratory behavior, but with key differences in the affected fold. The presence of the polyp adds mass and increases effective stiffness, reducing vibration amplitude and slightly lowering the frequency of the fold containing the lesion (left) in comparison to the opposing fold (right). Therefore, the VFP introduces asymmetric, asynchronous vibration, similar to clinical findings in [5]. Figure 8 further illustrates higher deformation and von Mises stress in the affected fold (left) compared to the corresponding fold in the case when VFP is not present, consistent with mass-induced frequency reduction. Additionally, the right fold in the lesion case, though lesion-free, displays increased displacement due to coupling effects with the impaired fold. Namely, tissue deformation, and therefore stress values, are greater when a VFP is present due to the mass-loading effect of the lesion on the VF dynamics. While the natural frequency of a vibrating system is inversely proportional to its mass, vibration amplitude tends to increase with mass, allowing the tissue to experience larger displacements under the same driving force. Thus, the lesion alters the vibratory pattern by lowering the frequency and increasing the extent of tissue oscillation compared to the healthy case, aligning with observations for abnormal VF vibration in the presence of a polyp in [28] and a nodule in [27], as well as experimental results in [16].

The findings suggest that lesions alter airflow patterns, VF vibration, and phase symmetry, which are critical factors for vocal quality and function, and increase tissue mechanical stresses that may contribute to disease progression and voice fatigue. While one contribution of this study is to capture overall glottal flow features and investigate the phenomenological changes in flow patterns induced by the lesion, quantitative analyses of vortex structures caused by the lesion and inking turbulence metrics to clinical energy loss is an important direction for future work. Given its size and position, the polyp may bridge the glottal gap and partially occlude the glottal flow, resulting in a more significant contribution to FSI features. Future research will investigate the effects of lesion morphology, including anatomical placement, to compare how obstruction modifies the trends identified in this study. Conducting a parametric analysis to quantify the effects of lesion mechanical properties on VF vibration characteristics, particularly by varying polyp stiffness systematically, is a promising direction for subsequent studies. Such an analysis would provide deeper mechanistic insights into the mechanical role of VFPs and could inform clinical management strategies. Additionally, establishing correlations between simulated biomechanical outputs (e.g., stress distributions) and clinical indicators, and extending these findings toward surgical planning and translational applications, is a natural extension of this work. However, such efforts require patient-specific datasets and clinical cohorts that lie beyond the scope of the present FSI modeling framework. This, therefore, remains an important direction for further investigation.

## Figures and Tables

**Table 1:** Parameters for larynx, VF tissue, and lesion domains and their respective values.

Domain	Parameter Description	Parameter Value
Vocal tract (larynx) $\Omega_{a}$	Density $\rho_{a}$	1.185 kg/m^3^
	Dynamic viscosity $\mu_{a}$	1.83 × 10^−5^ kg/m.s
	Minimum width of glottal gap	0.02 cm
VF body tissue $\Omega_{b}$	Density $\rho_{b}$	1070 kg/m^3^
	Poisson ratio $\nu_{b}$	0.45
	Young’s modulus $E_{b}$	40 kPa
	VF length	1.1 cm
	VF thickness	0.9 cm
	VF depth	1.0 cm
VF cover tissue $\Omega_{c}$	Density $\rho_{c}$	1070 kg/m^3^
	Poisson ratio $\mu_{c}$	0.45
	Young’s modulus $E_{c}$	10 kPa
	VF cover layer thickness	0.1 cm
VFP lesion $\Omega_{l}$	Density $\rho_{l}$	1300 kg/m^3^
	Poisson ratio $\nu_{l}$	0.45
	Modulus of elasticity $E_{l}$	5000 kPa
	Lesion length $L_{l}$	0.37 cm
	Lesion diameter $D_{l}$	0.15 cm

## Data Availability

The article contains all the data supporting the findings.
